# A Comparison of the Effects of the GLP-1 Analogue Liraglutide and Insulin Glargine on Endothelial Function and Metabolic Parameters: A Randomized, Controlled Trial Sapporo Athero-Incretin Study 2 (SAIS2)

**DOI:** 10.1371/journal.pone.0135854

**Published:** 2015-08-18

**Authors:** Hiroshi Nomoto, Hideaki Miyoshi, Tomoo Furumoto, Koji Oba, Hiroyuki Tsutsui, Arina Miyoshi, Takuma Kondo, Kenichi Tsuchida, Tatsuya Atsumi, Naoki Manda, Yoshio Kurihara, Shin Aoki, SAIS Study Group

**Affiliations:** 1 Division of Rheumatology, Endocrinology and Nephrology, Hokkaido University Graduate School of Medicine, Sapporo, Japan; 2 Department of Cardiovascular Medicine, NTT East Japan Sapporo Hospital, Sapporo, Japan; 3 Department of Cardiovascular Medicine, Hokkaido University Graduate School of Medicine, Sapporo, Japan; 4 Department of Biostatistics, School of Public Health, Graduate School of Medicine, The University of Tokyo, Tokyo, Japan; 5 Interfaculty Initiative in Information Studies, The University of Tokyo, Tokyo, Japan; 6 Manda Memorial Hospital, Sapporo, Japan; 7 Kurihara Clinic, Sapporo, Japan; 8 Aoki Clinic, Sapporo, Japan; University of Tolima, COLOMBIA

## Abstract

**Objectives:**

GLP-1 improves hyperglycemia, and it has been reported to have favorable effects on atherosclerosis. However, it has not been fully elucidated whether GLP-1 is able to improve endothelial function in patients with type 2 diabetes. Therefore, we investigated the efficacy of the GLP-1 analogue, liraglutide on endothelial function and glycemic metabolism compared with insulin glargine therapy.

**Materials and Methods:**

In this multicenter, prospective randomized parallel-group comparison study, 31 diabetic outpatients (aged 60.3 ± 10.3 years with HbA1c levels of 8.6 ± 0.8%) with current metformin and/or sulfonylurea treatment were enrolled and randomly assigned to receive liraglutide or glargine therapy once daily for 14 weeks. Flow mediated dilation (FMD), a comprehensive panel of hemodynamic parameters (Task Force Monitor), and serum metabolic markers were assessed before and after the treatment period.

**Results:**

A greater reduction (worsening) in %FMD was observed in the glargine group, although this change was not statistically different from the liraglutide group (liraglutide; 5.7 to 5.4%, glargine 6.7 to 5.7%). The augmentation index, C-peptide index, derivatives of reactive oxygen metabolites and BMI were significantly improved in the liraglutide group. Central systolic blood pressure and NT-proBNP also tended to be improved in the liraglutide-treated group, while improvements in HbA1c levels were similar between groups. Cardiac index, blood pressure and most other metabolic parameters were not different.

**Conclusions:**

Regardless of glycemic improvement, early liraglutide therapy did not affect endothelial function but may provide favorable effects on beta-cell function and cardioprotection in type 2 diabetics without advanced atherosclerosis.

**Trial Registration:**

UMIN Clinical Trials Registry System as trial ID UMIN000005331.

## Introduction

Type 2 diabetes is one of the major causes of atherosclerosis and an independent risk factor of cardiovascular events [1]. Indeed, it has been reported that the prevalence of coronary and peripheral artery disease is 2- to 4-fold higher, and stroke risk was also 2-fold higher in overt type 2 diabetic patients [2–4]. To prevent these atherosclerotic events, it is important to detect and intervene early in the development of atherosclerosis. Recently, endothelial cell dysfunction has been shown to precede endothelial thickening and atheroma development, and has been reported to be an important predictor of cardiovascular events also in type 2 diabetic patients [5, 6]. Moreover, they are used for therapeutic surrogate parameters of the early phase of atherosclerosis because of their plasticity. Flow-mediated dilation of the brachial artery (FMD) reflects endothelial nitric oxide (NO) bioavailability and is widely used as a marker for early atherosclerosis [7, 8]. Impaired FMD is associated with type 2 diabetes independent of glucose levels and may, in part, explain the increased cardiovascular risk in this patient population [9]. Therefore it is important that diabetic therapies achieve glycemic control and maintain/improve FMD to prevent the development of vascular complications. GLP-1 mimetics are a recently approved treatment strategy for improving glycemic control and lowering hemoglobin A1c (HbA1c) in type 2 diabetics. However, various additional health benefits beyond glucose control are also expected with GLP-1 therapy including protection from macro-vascular complications. For example, administration of GLP-1 improved endothelium-dependent vasodilatation in some rodent models [10, 11], and acute native GLP-1 infusion ameliorated endothelial and cardiac dysfunction in diabetic patients [12]. To date, a limited number of clinical trials have investigated the effects of commercially available GLP-1 analogues on endothelial function, and their results are inconsistent [12–15]. One of the possible reasons for theses varying results may be the difficulty associated with consistently assessing FMD due to technical and environmental factors. Therefore, the goal of the current study was to assess the effects of long-term treatment with the GLP-1 analogue Liraglutide on endothelial in patients with type 2 diabetes using a multicenter, prospective randomized parallel-group comparison study design.

## Materials and Methods

### Study population

The study enrolled 31 subjects with type 2 diabetes and adequately controlled blood pressure and plasma lipids. The patients were enrolled from 7 medical service units located in Sapporo city (SAIS Study Group). The inclusion criteria were patients with type 2 diabetes who were treated with or without metformin, sulphonylurea and/or DPP-4 inhibitor, aged 20–75 years, and had inadequate glucose control (defined as HbA1c between 7.4 to 10.5%). Patients who were diagnosed as having atherosclerotic diseases (angina, myocardial infarction, cerebral infarction and peripheral arterial disease), were currently receiving insulin therapy, were pregnant women, had a persistent elevation of their serum transaminase levels or had renal dysfunction were excluded.

### Protocol

This was a multicenter, open-labeled prospective randomized, parallel-group comparison study. Following enrollment, all individuals visited the Hokkaido University Hospital for measurement of a comprehensive panel of hemodynamic parameters (Task ForceMonitor), Endo-PAT, and serum metabolic markers. At the same time, FMD was performed by a well-trained technician who was blinded to the study to minimize the introduction of potential confounding variables given the sensitive nature of FMD analysis. The primary endpoint of the study was extent of change in FMD, and sample size was calculated using the assumption that liraglutide would improve FMD at least 2.0% based on previous studies using glimepiride and pioglitazone [16], or continuous infusion of GLP-1 [12]. It was determined that 13 patients were needed for each group to detect a significant difference with 80% power and statistical significance of 5%. Secondary endpoints were changes of metabolic parameters and surrogate markers of β cell function.

Patients were randomly assigned to receive once daily liraglutide (0.3 mg, 0.6 mg for 7 days followed by 0.9 mg/day) or glargine according to their age and basal FMD using computer software. All patients were encouraged to continue diet and exercise therapy during the study. Treatment was performed at each respective medical care center for 14 weeks, then endothelial function and serum biomarkers were measured again at the end of the study at Hokkaido University Hospital using the same parameters as at study entry. The subject enrollment period was from March 2011 to September 31, 2013 and the last subject completed the study in December of 2013.

### Flow-mediated dilatation and Taskforce Monitor

Endothelial function was evaluated using FMD of the brachial artery, according to published guidelines [17–19]. Briefly, the study was performed in the morning following an overnight fast and before taking any medications. Participants were to abstain from smoking, caffeine and antioxidant vitamins on the study day and received only drinking water prior to FMD assessment. Patients were kept in a supine position for at least 15 min in a quiet, temperature-controlled room (23°C to 26°C), then baseline FMD was measured using the brachial artery of their right arm. After 5min of suprasystolic compression (50mmHg over the systolic blood pressure) of the right forearm, the cuff was deflated and FMD was measured again. %FMD is expressed as percentage change from baseline to peak dilatation. All FMD measurements were made at a single location (Hokkaido University Hospital) by the same well-qualified technician that was blinded to the treatment groups.

After FMD measurement, beat to beat continuous finger BP, low frequency/high frequency ratio of R-R interval (LF/HF-RRI), cardiac index and total peripheral resistance index (TPRI) were measured noninvasively using a Task Force Monitor system (CNSystem, Austria) and reactive hyper index (RHI) was also measured by Endo-PAT (Itamar Medical Ltd., Cesarea, Israel).

### Biochemical analysis

For fasting serum analysis, collected blood samples were immediately placed on ice, centrifuged at 4°C, and the isolated supernatant frozen until measurement. LDL-cholesterol levels were calculated using the Friedewald formula. Plasma adiponectin, high-sensitivity C-reactive protein (hs-CRP), glucagon, TNF-α, total PAI-1, NT-proBNP, proinsulin and super oxide dismutase (SOD) were measured by latex agglutination, nephelometry, RIA, ELISA, latex photometric immunoassay, ECLIA, RIA, and improved nitrite ion method,, respectively (SRL, Inc, Tokyo, Japan).

### Evaluation of relevant factors

Homeostasis model assessment of insulin resistance (HOMA-IR) was calculated from fasting plasma glucose and insulin concentrations using this formula; (fasting plasma glucose (mg/dl) x insulin concentration (μU/ml)) /405. Proinsulin/C-peptide ratio and C-peptide index were calculated for assessment of β-cell function. C-peptide index was assessed using following formula: C-peptide index = [CPR (ng/ml) / Fasting plasma glucose (mg/dl)] x 100.

### Derivatives of reactive oxygen metabolites (d-ROM) and biological anti-oxidant potential (BAP) test

The levels of d-ROM and BAP were measured as an index of production of reactive oxygen species and an index of anti-oxidant potential using a free radical elective evaluator (the Free system, Diacron, Italy) according to previous reports [20]. Briefly, 20 μl of serum was mixed with acetic acid buffer (pH 4.8) in a pipet to stabilize the hydrogen ion concentration. Bivalent and trivalent iron from the serum proteins in acidified medium blood proteins in the acetic acid buffer and worked to catalyze hydroperoxide groups in the serum to become free alkoxy and peroxy radicals. Then these mixtures were transferred into cuvettes containing the colorless chromogen N, N-diethyl-p-phenylenediamine which turns to magenta if oxidized by free radicals into radical cations. The intensity of the magenta color reflects the concentration of hydroperoxides in the serum. After incubation for 5 min at 37°C, the intensity of the magenta color of cuvettes was measured by photometry (505 mm) after centrifuging for 1 min to measure the concentration of hydroperoxide. The values of hydroperoxides were shown as arbitrary units (U. CARR) [21].

BAP was simultaneously measured. The trivalent FeCl_3_ salt turns red due to the action of trivalent Fe^2+^ ions caused by anti-oxidant action. In theory, the anti-oxidant potential of serum can be evaluated by measuring the degree of decolorization using a photomer. In practice, the amount of trivalent iron from 10 μl of serum that is deoxidized in 5 min was measured in units of μmol/l [21].

### Statistical Analysis

Primary endpoint was change in FMD after 14 weeks. Secondary endpoints were several endothelial function and clinical and biochemical parameters (central systolic blood pressure, augmentation index, cardiac index, LF/HF-RRI, TPRI, RHI, FPG, HbA1c, Proinsulin/CPR ratio, C-peptide index, Glucagon, LDL-cholesterol, HDL-cholesterol, Triglyceride, Adiponectin, SOD activity, total PAI-1, NT-proBNP, TNF-α, hs-CRP, urinary albumin excretion, d-ROMs and BAP).

Based on previous evidence [12, 16], power calculations determined that a sample size of 13 individuals per group was required to have at least 80% power to detect a mean change in FMD of 2.0% (SD = 2.0) in the liraglutide group and 0.1% (SD = 1.0) in the glargine group. To account for potential loss of subjects over the 14-weeks study, a sample size of 15 patients for each group (30 patients in total) was established.

Results were expressed as mean ± SD or median. Differences of baseline characteristics between two groups were assessed by Welch’s *t*-test or Mann-Whitney U test for continuous variables and Fisher’s exact test for categorical variables. The Kolmogorov—Smirnov test for normality was used to determine the appropriate statistical test for the continuous variables. As the primary analysis, the effects of liraglutide compared to glargine on FMD were assessed by ANCOVA adjusted for baseline FMD. Mean changes between baseline and post-treatment of endothelial and metabolic parameters in both groups were analyzed descriptively as the secondary analyses. We also employed paired *t*-test or Wilcoxon signed test without the adjustment of the multiplicities because these secondary analyses were exploratory results. The Relationship between changes in %FMD and other metabolic variables such as body mass index (BMI), HbA1c and β-cell function were assessed using a Pearson’s correlation and Spearman’s rank-correlation analysis. A p-value <0.05 was considered statistically significant. Data were analyzed using Ekuseru-Toukei 2012 (Social Survey Research Information, Tokyo, Japan).

### Ethics statement

This study protocol was reviewed and approved by the institutional review board of Hokkaido University and written consent was obtained from all participants. This study has been registered in the UMIN Clinical Trials Registry System as trial ID UMIN000005331.

## Results

### Baseline characteristics

**Fig 1:**
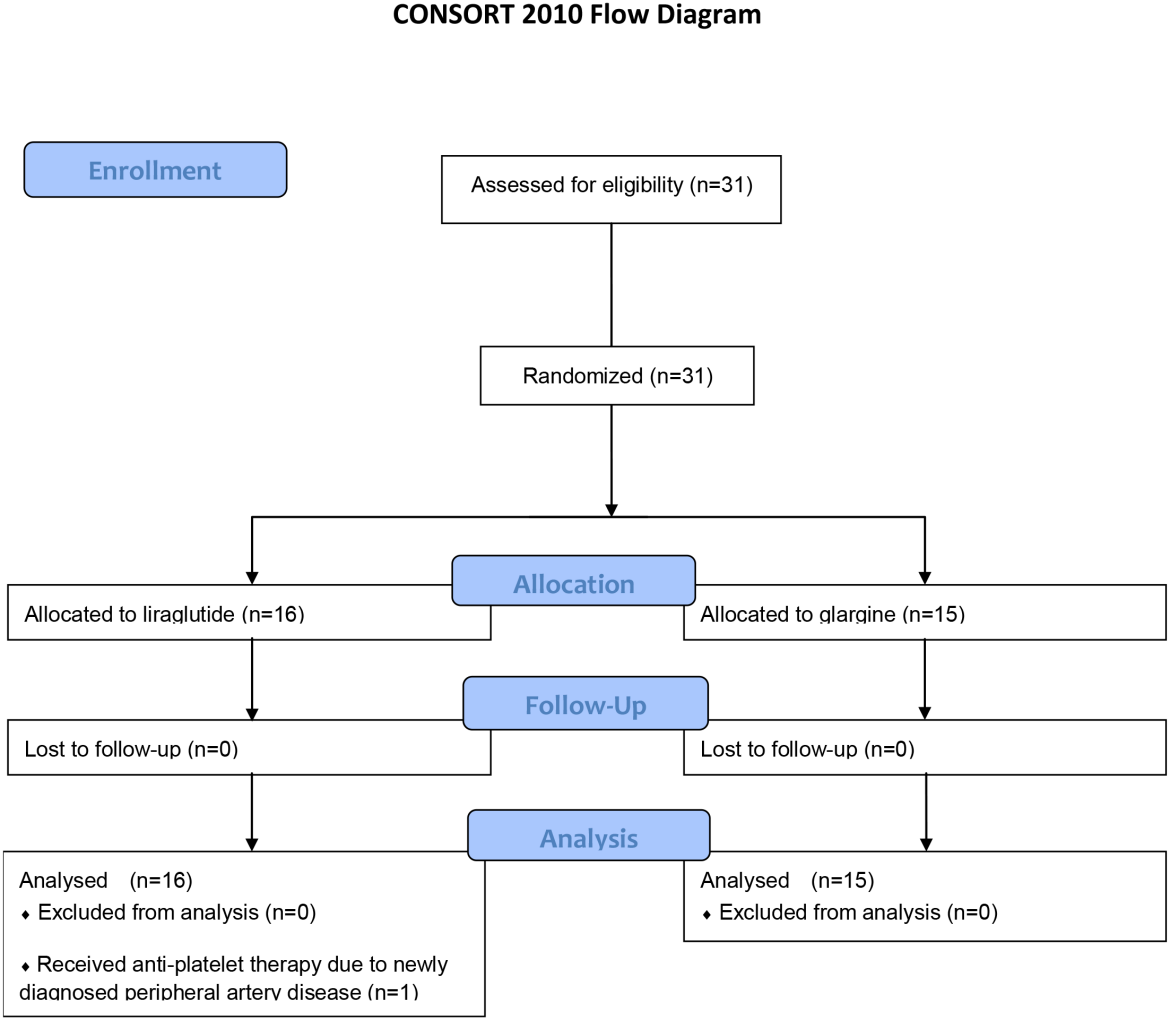
CONSORT Flowchart Participant Flow and Recruitment.

Thirty-one patients were enrolled, completed the initial examination and were randomized to each group. All patients completed the study although one patient in the liraglutide group was diagnosed with peripheral artery disease during the study period and started on an anti-platelet agent (Fig 1). The nineteen men and twelve women were an average age of 60.3 ± 10.3 years with HbA1c levels of 8.6 ± 0.8%. Other baseline characteristics of each group are shown in Table 1. There were no differences between the two groups in BMI, blood pressure, biological parameters, prevalence of current smoking, diabetic complications or proportion of oral hypoglycemic agents, renin-angiotensin-system blockers and statins. Liraglutide and glargine were well-tolerated throughout the study. Since the test of normality was rejected for BMI, low frequency/high frequency ratio of R-R interval, C-peptide, Proinsulin/C-peptide ratio, C-peptide index, Triglyceride, Adiponectin, SOD activity, total PAI-1, NT-proBNP, hsCRP, 8-OHdG and u-albumin, a non-parametric test was used for the comparison of these variables. All raw data were listed in S1 and S2 Tables.

**Table 1:** Clinical characteristics of the study population.

Variables	Liraglutide (n = 16)	Glargine (n = 15)	*P* value
**Age (yr)**	61.1 ± 8.3	59.5 ± 12.3	0.69
**Male gender (%)**	75.0	46.7	0.15
**Body mass index (kg/m** ^**2**^ **)**	26.6 (23.6 to 29.9)	25.8 (24.2 to 28.2)	0.71*
**Flow mediated dilatation (%)**	6.0 ± 2.6	6.7 ± 3.3	0.39
**FPG (mM/L)**	9.8 ± 2.1	9.7 ± 2.1	0.84
**Hemoglobin A1c (%)**	8.6 ± 0.8	8.7 ± 0.8	0.66
**SBP (mmHg)**	136.0 ± 14.9	126.4 ± 14.2	0.08
**DBP (mmHg)**	77.9 ± 11.1	73.0 ± 9.5	0.19
**LDL-cholesterol (mg/dl)**	109.5 ± 23.6	109.7 ± 20.6	0.98
**Current smokers (%)**	37.5	53.3	0.48
**Hypertension (%)**	68.8	53.3	0.47
**Dyslipidemia (%)**	62.5	80.0	0.43
**Angiotensin-converting enzyme inhibitors/ angiotensin II receptor blockers (%)**	60.0	26.7	0.15
**Statin (%)**	43.8	46.7	1.00
**Diabetic medication: metformin (%)/ sulfonylurea (%)/ DPP-4 inhibitor (%)**	100/87.5/43.8	93.3/80.0/66.7	0.48/0.65/0.29
**Diabetic nephropathy (%)**	31.3	40.0	0.72

FPG, fasting blood glucose; SBP, systolic blood pressure; DBP, diastolic blood pressure; DPP-4, dipeptidyl peptidase-4. Values are mean　±　SD or median (interquartile). *P* value of liraglutide vs glargine treated groups.

* Mann—Whitney U test was applied to body mass index.

### Endothelial functions and atherosclerosis

**Fig 2:**
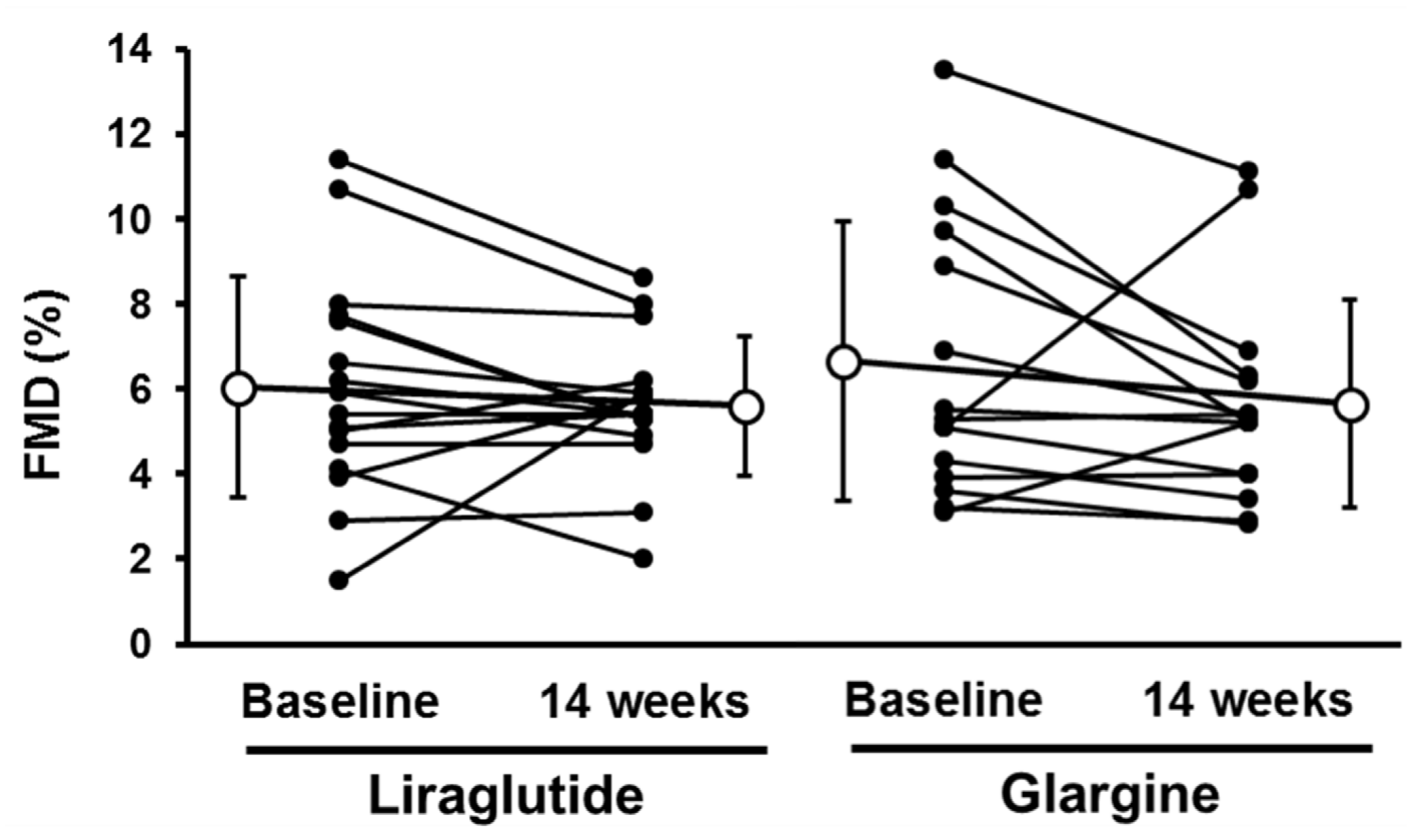
Comparison of individual changes of FMD before and after administration of liraglutide and glargine. White circles are mean ± SD.

Data in Fig 2 appear to indicate that a greater reduction (worsening) in %FMD occurred after treatment with glargine as compared to liraglutide, however change in %FMD was not statistically different between the liraglutide and glargine groups (Table 2). The changes in reactive hyperemia index (RHI) using Endo-PAT also were not different between these groups (Table 3). Liraglutide treatment significantly improved the augmentation index calculated from the blood pressure of the radial artery (p = 0.04), moreover centric systemic blood pressure tended to be lower (p = 0.07), although changes in systemic and diastolic blood pressure were not different in either groups. Changes in cardiac index, total peripheral resistance index and low frequency/high frequency ratio of R-R interval were similar in both groups (Table 3).

**Table 2:** Comparison of changes in %FMD before and after treatment in each group.

	Liraglutide (n = 16)	Glargine (n = 15)	*P* value
**FMD (%)**			
**Baseline**	6.04 ± 2.62	6.65 ± 3.30	
**14 weeks**	5.60 ± 1.64	5.65 ± 2.46	
**ΔFMD (%)**	-0.44 ± 1.90	-1.01 ± 2.63	0.50
	(-1.46 to 0.57)	(-2.46 to 0.45)	
**Adjusted ΔFMD (95% CI)**	-0.61 (-1.44 to 0.23)	-0.83 (-1.69 to 0.03)	0.70

Values are expressed as mean ± SD or the least square means (95% CI). The least square means were calculated by ANCOVA adjusted for baseline FMD.

**Table 3:** Comparison of the effects on endothelial function and clinical and biochemical parameters between liraglutide and glargine.

Variables	Liraglutide (n = 16)	Glargine (n = 15)
	Mean change from baseline (95% CI)	Mean change from baseline (95% CI)
^†^ **BMI (kg/m** ^**2**^ **)**	-0.48 (-0.84 to -0.11)	-0.11 (-0.31 to 0.09)
**SBP (mmHg)**	0.3 (-11.9 to 12.5)	4.9 (-5.1 to 14.9)
**DBP (mmHg)**	0.6 (-5.9 to 7.2)	2.9 (-4.3 to 12.9)
**Cardiovascular functions**		
* **cSBP (mmHg)**	-8.5 (-17.8 to 0.9)	3.4 (-3.1 to 9.9)
* **Augmentation index**	-5.3 (-10.4 to -0.2)	1.3 (-3.3 to 5.9)
**Cardiac index**	0.09 (-0.17 to 0.35)	0.06 (-0.12 to 0.25)
**TPRI**	-221.3 (-564.8 to 122.2)	-141.1 (-500.2 to 218.1)
* ^†^ **LF/HF ratio of RRI**	1.4 (-0.1 to 2.8)	0.6 (-2.2 to 3.4)
** **RHI**	0.21 (-0.11 to 0.54)	0.11 (-0.21 to 0.44)
**Biochemical parameters**		
**FPG (mM/l)**	-1.8 (-2.6 to -1.0)	-2.0 (-3.1 to -0.9)
**Hemoglobin A1c (%)**	-1.20 (-1.54 to -0.86)	-0.65 (-1.34 to 0.03)
^†^ **CPR (ng/ml)**	0.06 (-0.34 to 0.46)	-0.28 (-0.48 to -0.08)
^†^ **Proinsulin / CPR ratio**	-1.7 (-4.5 to 1.1)	7.2 (0.0 to 14.4)
^†^ **C-peptide index**	0.38 (0.08 to 0.68)	-0.01 (-0.11 to 0.10)
**Glucagon (pg/ml)**	9.5 (-2.5 to 21.5)	0.0 (-9.3 to 9.3)
**LDL-cholesterol (mg/dl)**	-6.5 (-13.3 to 0.3)	-2.6 (-9.7 to 4.4)
**HDL-cholesterol (mg/dl)**	0.9 (-2.1 to 4.0)	1.9 (-1.9 to 5.6)
^†^ **Triglyceride (mg/dl)**	-1.0 (-24.5 to 22.5)	-33.1 (-64.4 to -1.9)
^†^ **Adiponectin (μg/ml)**	0.17 (-0.15 to 0.49)	0.31 (-0.24 to 0.87)
^†^ **SOD activity (U/ml)**	0.13 (-0.21 to 0.47)	0.39 (-0.22 to 1.00)
^†^ **Total PAI-1 (ng/ml)**	-7.2 (-17.2 to 2.8)	3.1 (-1.5 to 7.8)
^†^ **NT-proBNP (pg/ml)**	-13.6 (-25.8 to -1.4)	6.1 (-10.9 to 23.1)
**TNF-α (pg/ml)**	0.03 (-0.41 to 0.46)	-0.11 (-0.32 to 0.10)
^†^ **log hsCRP (ng/ml)**	-0.05 (-0.32 to 0.23)	0.07 (-0.27 to 0.40)
^†^ **u-albumin (g/g.Cre)**	-0.09 (-0.22 to 0.03)	-0.04 (-0.14 to 0.06)
**Oxidative stress**		
**d-ROMs**	-31.5 (-52.9 to -10.1)	-2.7 (-46.3 to 41.0)
**BAP**	156.0 (-560.4 to 872.3)	77.7 (-68.7 to 224.0)

* Data were obtained in 30 patients (Liraglutide N = 15, Glargine N = 15).

** Data were obtained in 23 patients (Liraglutide N = 12, Glargine N = 11)

BMI, body mass index; SBP, systolic blood pressure; DBP, diastolic blood pressure; FMD, flow-mediated dilatation; cSBP, centric systolic blood pressure; TPRI, total peripheral resistance index; LF, low frequency; HF, high frequency; RRI, R-R interval; RHI, reactive hyperemia index; FPG, fasting blood glucose; SOD, superoxide dismutase; PAI-1, plasminogen activator inhibitor-1; NT-proBNP, N terminal prohormone of brain natriuretic peptide; TNF-α, tumor necrosis factor alpha; hsCRP, high-sensitivity C-reactive protein; d-ROMs, reactive oxygen metabolites-derived compounds; BAP, biological antioxidant potential. Values are mean change from baseline mean (95%CI).

^†^ Values were analyzed using Wilcoxon signed test since the normality was rejected in these variables.

### Glycemic control and metabolic parameters

At the end of this study, glycemic control, including HbA1c and fasting plasma glucose, was improved to the same extent in both groups (Table 3). However, the C-peptide index, which reflects beta-cell function, was significantly improved in the liraglutide treated group only (p = 0.03) (Table 3). Plasma glucagon and adiponectin levels were not significantly changed. In regards to the fasting lipid profile, LDL-cholesterol, HDL-cholesterol, triglyceride, RLP-cholesterol and other apolipoproteins were unchanged in both groups. Liraglutide administration tended to lower NT-proBNP (p = 0.07), which was not observed in the glargine group. Moreover, oxidative stress, measured by d-ROMs, was significantly diminished by liraglutide (p<0.001), but not glargine.

There were significant negative correlations between the degree of changes in %FMD and beta-cell function in both treatment groups, while %FMD and NT-proBNP were negatively correlated only in the glargine group (Table 4). However, improvements in HbA1c or other parameters were not significantly correlated with change in %FMD.

**Table 4:** Relationship between the changes in %FMD and other metabolic parameters pre- and post-treatment with liraglutide or glargine.

Variables	r *	r **	*P* value for the Spearman’s rank-correction	r *	r **	*P* value for the Spearman’s rank-correction
Medication	Liraglutide			Glargine		
**BMI**	0.023	0.037	0.892	-0.220	-0.382	0.160
**HbA1c**	-0.301	-0.245	0.361	-0.289	-0.211	0.450
**C-peptide index**	0.119	0.135	0.617	-0.581	-0.389	0.152
**Proinsulin /CPR index**	-0.572	-0.615	0.011	-0.268	-0.332	0.227
**cSBP**	-0.059	-0.005	0.985	-0.047	-0.383	0.159
**Augmentation index**	-0.183	-0.150	0.593	-0.099	-0.167	0.551
**NT-proBNP**	-0.273	-0.246	0.359	-0.665	-0.536	0.039
**total-PAI1**	-0.123	-0.052	0.848	-0.311	-0.242	0.384
**adiponectin**	-0.011	-0.030	0.912	0.028	0.054	0.850
**LDL-cholesterol**	-0.307	-0.107	0.694	-0.133	-0.136	0.630
**d-ROMs**	-0.419	-0.220	0.414	0.130	0.043	0.880

BMI, body mass index; CPR, C-peptide; cSBP, centric systolic blood pressure; PAI-1, plasminogen activator inhibitor-1; d-ROMs, reactive oxygen metabolites-derived compounds.

* Correlation coefficient for Pearson’s collection analysis,

** correlation coefficient for Spearman’s rank-correction.

## Discussion

In this study, we aimed to clarify the efficacy of exogenous administration of a GLP-1 analogue or insulin on endothelial function, independent of glucose lowering effects. This trial demonstrated that treatment of type 2 diabetic patients with the GLP-1 analogue liraglutide had no effect on %FMD when compared to glargine. These data are in contrast to previous studies where native GLP-1 administration improved glucose metabolism and increased %FMD in diabetic subjects [12]. In this trial, liraglutide reduced HbA1c levels from baseline similar to glargine treatment (-1.2% vs -0.7%, p = 0.14), but improvement in %FMD was not observed in either group. Calculated %FMD has been reported to be affected by many confounding factors, for example patients’ background (gender, age, obesity, heart rate and smoking) [22], conditions (air temperature, mental/physical stress) [23–25], medications such as angiotensin II receptor blockers [26], statins [27] and some anti-diabetic agents [16, 28]. However, prevalence of these potential confounders was not different between groups and when adjusted for baseline %FMD, there remained no statistical difference between groups. Moreover, one of the strengths of the current study was that measurement of FMD was performed by a well-qualified technician that was blinded to the treatment groups. While some previous clinical reports have shown favorable effects of GLP-1 itself or a GLP-1 receptor agonist on endothelial function [12, 13, 29, 30], many of these studies made comparisons to placebo treated groups where the potential effects of improved of blood glucose metabolism on endothelial function cannot be excluded. Moreover a recent meta-analysis also mentioned acute but not chronic GLP-1 based treatment may favorable effect FMD [31]. Furthermore, additional reported effects of GLP-1 include lowering of blood pressure and plasma lipids which also may independently influence endothelial function [13]. In the context of the present study, our results clearly demonstrated that liraglutide did not improve endothelial function measured by %FMD and Endo-PAT when compared to individuals treated with insulin glargine.

Secondary endpoint data from our study suggest possible beneficial effects of liraglutide on cardiovascular function when compared with insulin therapy. Liraglutide treatment reduced the augmentation index which is a known independent predictor of cardiovascular events [32] and is increased in patients with diabetes [33]. Because excessive cardiac load cause vascular remodeling and cardiac hypertrophy [34], this reduction may reflect cardioprotective effects of liraglutide. Recent studies have demonstrated that the endothelium does not possess the GLP-1 receptor [35, 36], suggesting that the beneficial effects of GLP-1 receptor agonist therapy on cardiovascular events and atherosclerosis may be explained by indirect mechanisms rather than direct action on endothelial cells.

Moreover, improvement of β-cell function was also observed in the liraglutide group despite a reduction in HbA1c similar to that with glargine. Many basic studies have demonstrated a protective effect of liraglutide on pancreatic β-cells [37, 38], but little is known about the in vivo effects of liraglutide on β-cell function or how they compare to that of insulin glargine. CPR index was used as a biomarker for β-cell function [39], and our data showed significant improvement in this surrogate marker with liraglutide treatment. Furthermore, liraglutide reduced oxidative stress as indicated by decreased d-ROMs. The d-ROMs test quantifies hydroperoxide level and provides an index for oxidative stress and reactive oxygen species (ROS). Given that ROS are known to oxidize LDL, denature and devitalize proteins and cause DNA injury, elevations in ROS could increase susceptibility to atherosclerosis and metabolic diseases including diabetes [40, 41]. In addition, overexpression of an anti-oxidant enzyme in cardiac mitochondria resulted in amelioration of not only oxidative stress but also mitochondrial dysfunction and hypertrophy of the myocardium after myocardial infarction [42]. Moreover, GLP-1 is reported to show anti-oxidant effects in pancreatic islet suppressing expression of nitric oxide synthase partly through the PKA pathway in pancreatic islets [43]. Pancreatic beta cells are sensitive to some oxidative stresses due to relatively low expression of anti-oxidant enzymes such as SOD, catalase and glutathione peroxidase [44]. Reduction of oxidative stress may, in part, contribute to the beneficial effects on both cardiac and beta cell function.

In conclusion, this is the first report of a direct comparison of liraglutide and glargine on endothelial function, and our study demonstrates that liraglutide has favorable effects on cardiovascular and pancreatic β-cell function as compared to glargine, while endothelial function did not differ between groups.

## Limitation

A potential limitation of our study was the half-life of liraglutide. The last dose of liraglutide was administered one day prior to assessment of FMD. The t_max_ and elimination half-life of liraglutide are reported to be 9 to 12 hours and 14 to 15 hours, respectively. Therefore, the plasma concentrations of liraglutide might have been too low to have a direct effect on FMD. Additional limitations include the small sample size, short study duration and the lack of double blinding. To resolve these potential issues, our findings need to be validated in a double-blind study, using a larger population over a longer period of time.

## Supporting Information

S1 Table All raw data in this study at 0 week. (XLSX)

S2 Table All raw data in this study at 14 weeks. (XLSX)

S1 Protocol Protocol of SAIS-2 Study. (DOCX)

S2 Protocol Protocol of SAIS-2 Study in Japanese. (DOC)

S1 CONSORT Checklist CONSORT 2010 checklist. (DOC)
