# Supplementary material for: A Comparison of the Effects of the GLP-1 Analogue Liraglutide and Insulin Glargine on Endothelial Function and Metabolic Parameters: A Randomized, Controlled Trial Sapporo Athero-Incretin Study 2 (SAIS2)
Source: PLoS One. 2015 Aug 18;10(8):e0135854. doi: 10.1371/journal.pone.0135854 (PMC4540491; doi:10.1371/journal.pone.0135854)
Supplement: S1 Protocol — (DOCX) [file pone.0135854.s003.docx]

**A Comparison of the effects of the GLP-1 Analogue Liraglutide and Insulin Glargine on Endothelial Function and Metabolic Parameters**

**Principal investigator**

Hideaki Miyoshi (Hokkaido University Hospital internal medicine II)

**Contributor**

Tomoo Frumoto (Department of Cardiovascular Medicine, Hokkaido University Hospital)

Ichizo Tsujino (Department of Medicine I, Hokkaido University Hospital)

Takuma Kondo (Department of Medicine II, Hokkaido University Hospital)

So Nagai (Department of Medicine II, Hokkaido University Hospital)

Yo Ohira (Department of Medicine I, Hokkaido University Hospital)

Osamu Nakagaki (Department of Medicine II, Hokkaido University Hospital)

Jun Takeuchi (Department of Medicine II, Hokkaido University Hospital)

Kyu-yong Cho (Department of Medicine II, Hokkaido University Hospital)

Manabu Noda (Department of Medicine II, Hokkaido University Hospital)

Kazuki Tajima (Department of Medicine II, Hokkaido University Hospital)

Hiraku Kameda (Department of Medicine II, Hokkaido University Hospital)

Hiroshi Nomoto (Department of Medicine II, Hokkaido University Hospital)

Nozomi Aikawa (Department of Medicine II, Hokkaido University Hospital)

Arina Miyoshi (Department of Medicine II, Hokkaido University Hospital)

Kohei Yamamoto (Department of Medicine II, Hokkaido University Hospital)

Chiho Yamamoto (Department of Medicine II, Hokkaido University Hospital)

Naohide Koyanagawa (Department of Medicine II, Hokkaido University Hospital)

January 25, 2013 version 7

**1． Background of the study**

Type 2 diabetes is one of the major causes of atherosclerosis and an independent risk factor of cardiovascular events^1)^. Indeed, it has been reported that the prevalence of coronary and peripheral artery disease is 2- to 4-fold higher, and stroke risk was also 2-fold higher in overt type 2 diabetic patients^2)-4)^. To prevent these atherosclerotic events, it is important to detect and intervene early in the development of atherosclerosis. Recently, endothelial cell dysfunction has been shown to precede endothelial thickening and atheroma development, and has been reported to be an important predictor of cardiovascular events also in type 2 diabetic patients ^5), 6)^. Moreover, they are used for therapeutic surrogate parameters of the early phase of atherosclerosis because of their plasticity. Flow-mediated dilation of the brachial artery (FMD) reflects endothelial nitric oxide (NO) bioavailability and is widely used as a marker for early atherosclerosis^7), 8)^. Impaired FMD is associated with type 2 diabetes independent of glucose levels and may, in part, explain the increased cardiovascular risk in this patient population^9)^. Therefore, it is important that diabetic therapies achieve glycemic control and maintain/improve FMD to prevent the development of vascular complications. GLP-1 mimetics are a recently approved treatment strategy for improving glycemic control and lowering hemoglobin A1c (HbA1c) in type 2 diabetics. However, various additional health benefits beyond glucose control are also expected with GLP-1 therapy, including protection from macro-vascular complications. For example, administration of GLP-1 improved endothelium-dependent vasodilatation in some rodent models ^10), 11)^, and acute native GLP-1 infusion ameliorated endothelial and cardiac dysfunction in diabetic patients^12)^. To date, a limited number of clinical trials have investigated the effects of commercially available GLP-1 analogues on endothelial function, and their results are inconsistent^12) -15)^.

Therefore, the goal of the current study was to assess whether long-term treatment with the GLP-1 analogue liraglutide can improve endothelial function in patients with type 2 diabetes using a multicenter, prospective randomized parallel-group comparison study design. We provide the first direct comparison of changes in endothelial function following chronic treatment with one of two functionally distinct glucose lowering therapies, liraglutide or insulin glargine.

**2． Purpose of this study**

We investigated the efficacy of the GLP-1 analogue, liraglutide on endothelial function and glycemic metabolism compared with insulin glargine therapy in the design of multicenter, prospective, non-blinded, randomized parallel-group comparison study.

**3． Summary of the study drug**

3.1 Insulin glargine

* Manufacture/distributor: Sanofi-Aventis Co., Ltd.

* Mechanism of action: a long-acting basal [insulin analogue](http://en.wikipedia.org/wiki/Insulin_analogue), given once daily to help control the [blood glucose](http://en.wikipedia.org/wiki/Blood_sugar) level of those with [diabetes](http://en.wikipedia.org/wiki/Diabetes). It consists of microcrystals that slowly release insulin, giving a long duration of action of 18 to 26 hours, with a "peakless" profile.

3.2 Liraglutide

* Production, a sales agency: Novo Nordisk Co., Ltd.

* Mechanism of action: a long-acting [glucagon-like peptide-1](http://en.wikipedia.org/wiki/Glucagon-like_peptide-1) [agonist](http://en.wikipedia.org/wiki/Agonist) [(GLP-1 agonist)](http://en.wikipedia.org/wiki/Glucagon-like_peptide-1_agonist), given once daily to reduces meal-related [hyperglycemia](http://en.wikipedia.org/wiki/Hyperglycemia) (for 24 hours after administration) by increasing [insulin](http://en.wikipedia.org/wiki/Insulin) secretion, delaying gastric emptying, and suppressing prandial [glucagon](http://en.wikipedia.org/wiki/Glucagon) secretion.

**4． The study patients and competent criteria**

Patients who fulfill all of these criteria are eligible; (1), (2) inclusion criteria and (3) exclusion criteria.

(1) The study patients

Patients with type 2 diabetes who were treated at the Hokkaido University Hospital, or otherinstitutions participating in this study.

(2) Inclusion criteria

(1) Age is 20 years or older at the time of consent.

(2) Inadequate HbA1c control, 7.4-10.5 % (NGSP), 7.0-10.0 % (JDS) at the agreed acquisition

(3) Patients receiving sulfonylurea, metformin, or a DPP-4 inhibitor alone or in combination in addition to a diet and exercise therapy.

(4) The patients who have adequate control of blood pressure and dyslipidemia.

(5) The patients who are able to understand the requirements of the study, and provide consent of their own free will.

(3) Exclusion criteria

(1) The patients who are treated with insulin therapy.

(2) The patients who are suffering serious liver damage, renal disease, or heart failure.

(3) The patients who underwent angina, myocardial infarction, cerebral infarction or arteriosclerosis obliterans.

(4) The patients who take more than three antihypertensive medications.

(5) The patients who takemore than two lipid lowering medications.

(6) The patients who have a history of hypersensitivity to insulin or GLP-1 receptor agonists.

(7) A pregnant woman, nursing mother or woman who may become pregnant.

(8) In addition, the patients whom a principal investigator judged to be inadequate as subjects.

**5． Method of the study**

(1) Study design

Multicenter, prospective, non-blinded, randomized, parallel-group comparison study.

(2) Study outline

(1) Participants received an initial screening (week 0) at the Department of Cardiovascular Medicine, Hokkaido University Hospital.

(2) The medication is to be started within a month after screening. A starting dose of liraglutide and insulin glargine was 0.3 mg/day and 4-8 unit/day, respectively.

(3) Glycemic control is assessed at each institution every 4-8 weeks.

(4) A final examination is carried out at the Department of Cardiovascular Medicine, Hokkaido University Hospital at 14 weeks (+/- two weeks).

| At registration | week 0 | | Every 4-8 weeks | 14 weeks |
| --- | --- | --- | --- | --- |
| Written consent | Screening before randomization and administration | |  | Final examination |
| SU, metformin and/or DPP4 inhibitor | Insulin glargine group | 4-8  Unit | appropriate increase and decrease | appropriate increase and decrease |
|  | liraglutide group | 0.3 mg | 0.9 mg | 0.9 mg |
| FMD | ● |  |  | ● |
| TBI | ● |  |  | ● |
| Taskforce monitor | ● |  |  | ● |
| Blood glucose  (HbA_1c_, FPG) | ● |  | ● | ● |
| Biomarker measurement | ● |  |  | ● |
| Clinical laboratory examinations | ● |  |  | ● |

● are mandatory.

(3) Injection method of the study agents

A starting dose of insulin glargine is 4-8 unit/day before sleep, and the dose is to be titrated according to the judgment of physicians in charge with the goal a HbA_1c_ below 6.9%. Liraglutide is started at a dose of 0.3 mg/day, and is increased to 0.6 mg/day after one week and then 0.9 mg/day at the end of the second week and maintained until the end of the study. DPP-4 inhibitors are discontinued at the beginning of the study.

(4) Concomitant medications

Patients who require the use of additional hypertension and/or dyslipidemia medications during the study period must be discontinued from the study.

(5) Method of registration and assignment

(1) Case registration

The physicians in charge completes the enrollment form and sends it to the study manager.

(2) Assignment of the study agents

The study manager receives the completed enrollment form which includes data from the screening examination performed at the Hokkaido University Hospital and randomly assigns study participants to either the insulin glargine group or liraglutide group, according to computerized randomization.

(6) Study period

Each participant is treated with insulin glargine or liraglutide for 14 weeks in each institution.

(7) Correspondence after the study

After this study, each physician is required to provide the most appropriate medical care to participants.

**6． Observational and analytical analyses**

Observational and analytical testing for the following items will be performed.

(1) Patients background: Patients initial, identification cord, age, sex, type of diabetes, height, weight, BMI, abdominal circumference, contraction of a disease period, complications, presence or absence of smoking, alcohol consumption, drug medication contents

(2) Blood pressure, pulse rate

(3) Laboratory study (fasting)

Urinalysis (sugar urinary, protein urine (qualitative analysis), the urinary albumin/ creatinine ratio), blood glucose (HbA_1c_, fasting plasma glucose), serum insulin, proinsulin, fasting serum lipids (total cholesterol, triglyceride, HDL-cholesterol, LDL-cholesterol, FFA, apoprotein, RLP-C), AST, ALT, γ-GTP, uric acid, BUN, serum creatinine, Na, K, Cl, hs-CRP, NT-proBNP

Adiponectin, total PAI-1, TNF - α, SOD, urinary 8-OHdG

In addition, for biomarker measurement use, we will collect an additional 10 ml of blood at the 0 and 14 week time points. If the agreement of subjects is obtained, we will store the remaining sample and may perform additional measurements when new biomarkers are found in the future.

(4) Toe brachial index (TBI)

(5) Flow mediated dilatation (FMD) ： FMD is influenced by blood glucose level and time of measurement. Thus, we will perform fasting FMD in the morning. We prohibit caffeine, smoking, or taking any vitamin supplements before the assessment, and perform FMD assessment in a thermo-regulated room according to guidelines. Oral medicines are discontinued only on the morning of the assessment day, and taken after testing.

(6) Taskforce monitor: Using a Taskforce monitor (CNSystems, Nihon Kohden) that is noninvasive cardiac activity measuring assembly, we evaluate the cardiac output, systemic vascular resistance, the autonomic nervous function. The study day requirements are the same as (5) because we perform (5) and (6) simultaneously.

**7． Expected benefit and disadvantage (potential side effect)**

(1) The expected benefit

We may choose more appropriate medication for individuals according to insights from this study. The results of this study may also contribute to future medical progress.

(2) The expected disadvantage

The details refer to an attached document of each agent. Hypoglycemia, constipation, and nausea are reported as frequent side effects for liraglutide, and hypoglycemia is reported for insulin glargine.

(1) FMD: Pain may occur by pressurizing a brachial artery with a cuff of the sphygmomanometer, but this testing is widely utilized in clinics and thepain is reported to be tolerable. However, FMD may be discontinued at the discretion of the attending doctor when pain is considered severe.

(2) Blood volumes will increase compared to standard clinical needs. These volumes are well below the suggested blood volume limits and will not affect the the care of the subjects.

**8． Endpoint**

(1) Primary outcome

We will compare the change in FMD between each group before and after agent administration.

(2) Secondary endpoint

Change in the following items between the treatment groups before and after the study.

(1) Blood glucose (HbA1c, fasting plasma glucose)

(2) Evaluation of pancreatic beta cell function

(3) The change of various measurements by TBI, Taskforce monitor

(4) Various blood test values, urine test value, biomarkers

**9． Discontinuation criteria for individual subjects**

(1) Correspondence at study cancellation

Based on the following criteria it may be decided that an individual should be discontinued from the study.

(2) Discontinuation criteria

(1) When a subject withdraws consent and/or refuses to participate in the study.

(2) When we identify individuals that were enrolled but do not meetthe inclusion criteria.

(3) When we judge a patient’s condition worsens due to studytreatment.

(4) When the continuation of the study is made difficult by the exacerbation of complications.

(5) When the continuation of the study is made difficult by an adverse event.

(6) When pregnancy becomes clear.

(7) When compliance is remarkably poor.

(8) The patients who do not adhere to diet and exercise therapy.

(9) When the study is terminated.

(10) When, for others reasons, the study person in charge judges the cancellation of the study to be suitable.

**10． The handling of adverse event development**

(1) Correspondence to subjects who suffer adverse events

When an adverse event occurs, the study person in charge will administer appropriate treatment promptly and document it in a medical record and a case report. Moreover, we accurately explain the cancellation of study and treatment of adverse event to the subjects.

(2) Report of serious adverse event

Serious adverse events are defined as follows.

1) Death, or complications that may lead to death

2) Hospitalization or extension of the length of stay

3) Sequela, or fear to lead to sequela

4) Effects to congenital disease or abnormality

The study person in charge has to immediately reports all serious adverse events suspected to be related to experimental treatments during study period to the head of the hospital through the clinical studies manager.

(3) Report of the important adverse event

Not applicable.

(4) Other adverse events

About other adverse events, the study person in charge describes it in a medical record and a case report appropriately.

**11． Changes such as study enforcement plans**

When we conduct the change of a study enforcement plan and the agreement explanation document of this study or revision, we require the approval of the voluntary clinical studies screening committee beforehand.

**12． Study amendment, cancellation, interruption, the end of the study**

(1) Amendments

When we amend/revise the study protocol and/or patient consent documents, we require the approval of the Hokkaido University Hospital independence clinical studies screening committee beforehand.

(2) Early termination, interruption of the study

The study person in charge may decide early termination of the study is appropriate in any of the following cases.

(1) When the recruitment of subjects is difficult and is judged to be extremely difficult to reach the planned number of cases.

(2) When the purpose of the study is accomplished before reaching during planned number of cases or a planned period.

(3) When we judge it is difficult to change our protocols if the screening committee propose us to drastically change our protocols.

When there is a recommendation for early termination by a screening committee, the study person in charge will terminate the study. The decision to terminate early or interrupt a study is documented along with the explaination and reported immediately to the head of the hospital.

(3) The end of the study

At the end of this study, the study person in charge submits a report of this study to the head of a hospital immediately.

**13． Study duration**

After approval (March, 2011) to December 31, 2013 (a registration final day: September 31, 2013)

**14． Goal number of cases and the setting backgrounds and statistical analysis method**

(1) Goal number: 30 cases in total (15 cases in each group)

Setting backgrounds

The detailed report about the effect on FMD with the administration of liraglutide is not found at present. According to the report of Papathanassiou K et al^13)^ which evaluates FMD changes six months after pioglitazone or glimepiride therapy, changes in %FMD were 2.0 % (standard deviation = 2.0) in the pioglitazone group compared with 0.1 % (standard deviation = 1.0) in the glimepiride group. Moreover, there was a report showing intravenous recombinant GLP-1 infusion for 105 minutes increased baseline FMD by almost 2-fold^14)^. We hypothesize that after 14 weeks of treatment the effect of liraglutide on FMD may be similar to the 2% (standard deviation = 2.0) change observed with pioglitazone and the effect of insulin will be equivalent to that of glimepiride (0.1% changes (standard deviation = 1.0)). From this assumption, it was determined that 13 patients were needed for each group to detect a significant difference with 80% power and statistical significance of 5%.

(2) Statistical analysis method

We will measure %FMD before and after treatment for all subjects and report a mean and standard deviation for each treatment group. We will compare changes in %FMD for each group using non-paired *t*-test. We will also assess the changes in the amount of %FMD using a multiple comparison testing for each change. In addition, we will determine the change in various biomarkers and β cell function for all subjects similarly and examine the association between the difference between groups and parameters in an exploratory fashion.

**15． Protective method of the consideration for the human rights of subjects and the personal information**

All people in charge of this study have to observe "Helsinki Declaration" (October, 2008 revision) and "the ethical guideline (July 31, 2008 revision, following clinical studies ethic guideline) about clinical studies".

Individual information and samples collected during this study will be assigned a unique sample number that is unrelated to the subject’s personal information. When we send information and samples to the associated organizations such as the study manager, we will use this unique identifying number to ensure that the subject’s personal information is not revealed. This numbering system will also be used when analyzing and reporting the results of the study. Information obtained from study subjects will not be used for anything other than the purposes of this study.

**16． Informed Consent**

The doctor in charge provides an informed consent document to the subjects and gives an oral explanation of the document and study and acquires a signed consent agreement with the free will of subjects.

If the protocol is amended in a way that influences the consent obtained from the subject, the doctor in charge has to give this new information to subjects immediately. At the same time, the doctor in charge has to confirm the intention of subjects, to continue their participation in this study, and obtain written consent again.

An informed consent document contains following components:

(1) Subjects are participating in this study of their own free will and don’t suffer a disadvantage because of nonparticipation. They are able to withdraw their consent to participate at any time.

(2) Significance (background), purpose, subject, method, duration of the study, planned subject number

(3) The benefit and disadvantage that are expected by participating in a study

(4) The methods about handling such as personal information, a conservation period and a material disposal.

(5) The handling about the announcement of results and a patent.

(6) The cost burden on subjects for participating in this study, a study source of funds and benefit reciprocity.

(7) Composition of research unit and consultation windows (contact information) of this study.

(8) Presence or absence of correspondence and compensation when a health hazard occurred in subjects.

**17． Correspondence and compensation to the health hazard of subjects**

In the case of a health hazard in subjects participating in the study, the doctor in charge treats appropriately. The compensation for the health hazard is carried out according to clinical studies ethic guideline. For death and severe disorder, we prepare for compensation money. For other health hazard, necessary treatment such as testing or treatment is to be provided in the medical service under health insurance of subjects.

**18． Costs burden on subjects**

The study person in charge is responsible for the costs associated with biomarker analysis and will utilize research funds from the department. Any other cost burden on subjects by participating in a study does not occur to be performed in a normal medical service under health insurance. In addition, we decide to provide 2,000 yen QUO CARD for every examination of arteriosclerosis (FMD, TBI, Taskforce monitor) enforcement for the purpose of burden on subjects’ reduction. The doctor in charge has to explain this to participants and allow subjects to decide if they wish to participate or not.

**19． The retention of records and publication of results**

The study person in charge has to save important documents (the copy of the application documents, a notice document from the head of a hospital, the consent form of various applications, reports, documents necessary to guarantee the reliability of other data or records) for three years after the completion or termination of the study. Subsequently personal information is carefully disposed.The results of the study will be presented at scientific meetings and in scientific journals.

**20． A funding source and benefit reciprocity**

This study is conducted using research funds fromthe department of the study person in charge. The handling of the benefit reciprocity examination is also conducted according to the rules of each institution. The study person in charge of the Hokkaido University Hospital has to report a necessary matter to the benefit reciprocity screening committee according to the rule of "benefit reciprocity management internal regulations affect clinical studies in the Hokkaido University Hospital" and should obtain the examination and approval.

**21． Study enforcement system**

This study is conducted with the following systems.

**Principal investigator**

Hideaki Miyoshi (Hokkaido University Hospital internal medicine II)

**Contributor**

Tomoo Frumoto (Department of Cardiovascular Medicine, Hokkaido University Hospital)

Ichizo Tsujino (Department of Medicine I, Hokkaido University Hospital)

Takuma Kondo (Department of Medicine II, Hokkaido University Hospital)

So Nagai (Department of Medicine II, Hokkaido University Hospital)

Yo Ohira (Department of Medicine I, Hokkaido University Hospital)

Osamu Nakagaki (Department of Medicine II, Hokkaido University Hospital)

Jun Takeuchi (Department of Medicine II, Hokkaido University Hospital)

Kyu-yong Cho (Department of Medicine II, Hokkaido University Hospital)

Manabu Noda (Department of Medicine II, Hokkaido University Hospital)

Kazuki Tajima (Department of Medicine II, Hokkaido University Hospital)

Hiraku Kameda (Department of Medicine II, Hokkaido University Hospital)

Hiroshi Nomoto (Department of Medicine II, Hokkaido University Hospital)

Nozomi Aikawa (Department of Medicine II, Hokkaido University Hospital)

Arina Miyoshi (Department of Medicine II, Hokkaido University Hospital)

Kohei Yamamoto (Department of Medicine II, Hokkaido University Hospital)

Chiho Yamamoto (Department of Medicine II, Hokkaido University Hospital)

Naohide Koyanagawa (Department of Medicine II, Hokkaido University Hospital)

**The study manager**

Hokkaido University Hospital internal medicine II

Person in charge: Hideaki Miyoshi

Contact information: North 15, West 7, Kita-ku, Sapporo, Hokkaido, 060-8638

Hokkaido University Hospital internal medicine II medical office, +81-11-706-5915

**Case assignment enforcement organization**

Translational Research and Clinical Trial Center in Hokkaido university hospital

Person in charge: Koji Ohba

Contact information: North 14, West 5, Kita-ku, Sapporo, Hokkaido, 060-8648

+81-706-7413

**Participating institution**

Aoki clinic, Ohtsuka eye hospital, Ohdori internal medicine clinic, Ogasawara clinic Sapporo hospital, Yuri Ono clinic, Clark hospital, Kurihara clinic, Soen central hospital, Hokkaido social insurance hospital, Manda memorial hospital

**22． References**

[1] Sarwar N, Gao P, Seshasai SR, et al. Diabetes mellitus, fasting blood glucose concentration, and risk of vascular disease: a collaborative meta-analysis of 102 prospective studies. Lancet. 2010; 375: 2215-2222

[2] Haffner SM, Lehto S, Ronnemaa T, Pyorala K, Laakso M. Mortality from coronary heart disease in subjects with type 2 diabetes and in nondiabetic subjects with and without prior myocardial infarction. The New England journal of medicine. 1998; 339: 229-234

[3] Newman AB, Siscovick DS, Manolio TA, et al. Ankle-arm index as a marker of atherosclerosis in the Cardiovascular Health Study. Cardiovascular Heart Study (CHS) Collaborative Research Group. Circulation. 1993; 88: 837-845

[4] Kimura K, Minematsu K, Kazui S, Yamaguchi T. Mortality and cause of death after hospital discharge in 10,981 patients with ischemic stroke and transient ischemic attack. Cerebrovascular diseases (Basel, Switzerland). 2005; 19: 171-178

[5] Beckman JA, Creager MA, Libby P. Diabetes and atherosclerosis: epidemiology, pathophysiology, and management. JAMA : the journal of the American Medical Association. 2002; 287: 2570-2581

[6] Calles-Escandon J, Cipolla M. Diabetes and endothelial dysfunction: a clinical perspective. Endocrine reviews. 2001; 22: 36-52

[7] Deanfield JE, Halcox JP, Rabelink TJ. Endothelial function and dysfunction: testing and clinical relevance. Circulation. 2007; 115: 1285-1295

[8] Hollenberg SM, Cinel I. Bench-to-bedside review: nitric oxide in critical illness--update 2008. Critical care (London, England). 2009; 13: 218

[9] Henry RM, Ferreira I, Kostense PJ, et al. Type 2 diabetes is associated with impaired endothelium-dependent, flow-mediated dilation, but impaired glucose metabolism is not; The Hoorn Study. Atherosclerosis. 2004; 174: 49-56

[10] Gaspari T, Liu H, Welungoda I, et al. A GLP-1 receptor agonist liraglutide inhibits endothelial cell dysfunction and vascular adhesion molecule expression in an ApoE-/- mouse model. Diabetes & vascular disease research : official journal of the International Society of Diabetes and Vascular Disease. 2011; 8: 117-124

[11] Han L, Yu Y, Sun X, Wang B. Exendin-4 directly improves endothelial dysfunction in isolated aortas from obese rats through the cAMP or AMPK-eNOS pathways. Diabetes research and clinical practice. 2012; 97: 453-460

[12] Nystrom T, Gutniak MK, Zhang Q, et al. Effects of glucagon-like peptide-1 on endothelial function in type 2 diabetes patients with stable coronary artery disease. American journal of physiology Endocrinology and metabolism. 2004; 287: E1209-1215

[13] Irace C, De Luca S, Shehaj E, et al. Exenatide improves endothelial function assessed by flow mediated dilation technique in subjects with type 2 diabetes: results from an observational research. Diabetes & vascular disease research : official journal of the International Society of Diabetes and Vascular Disease. 2013; 10: 72-77

[14] Kelly AS, Bergenstal RM, Gonzalez-Campoy JM, Katz H, Bank AJ. Effects of exenatide vs. metformin on endothelial function in obese patients with pre-diabetes: a randomized trial. Cardiovascular diabetology. 2012; 11: 64

[15] Hopkins ND, Cuthbertson DJ, Kemp GJ, et al. Effects of 6 months glucagon-like peptide-1 receptor agonist treatment on endothelial function in type 2 diabetes mellitus patients. Diabetes, obesity & metabolism. 2013; 15: 770-773

[16] Papathanassiou K, Naka KK, Kazakos N, et al. Pioglitazone vs glimepiride: Differential effects on vascular endothelial function in patients with type 2 diabetes. Atherosclerosis. 2009; 205: 221-226

[17] Nystrom T. The potential beneficial role of glucagon-like peptide-1 in endothelial dysfunction and heart failure associated with insulin resistance. Hormone and metabolic research = Hormon- und Stoffwechselforschung = Hormones et metabolisme. 2008; 40: 593-606
